# The role of collisional ionization in heavy ion acceleration by high intensity laser pulses

**DOI:** 10.1038/s41598-022-23148-2

**Published:** 2022-10-29

**Authors:** M. Afshari, S. Morris, L. D. Geulig, Z. M. Chitgar, P. Gibbon, P. G. Thirolf, J. Schreiber

**Affiliations:** 1 Fakultät für Physik, Ludwig-Maximilians-Universität München, 85748 Garching bei München, Germany; 2 Department of Physics, University of Warwick, Coventry, CV4 7AL UK; 3 Institute for Advanced Simulation, Jülich Supercomputing Centre, Forschungszentrum Jülich GmbH, 52425 Jülich, Germany; 4 Centre for Mathematical Plasma Astrophysics, Katholieke Universiteit Leuven, 3000 Leuven, Belgium

**Keywords:** Laser-produced plasmas, Plasma-based accelerators

## Abstract

We present here simulation results of the laser-driven acceleration of gold ions using the EPOCH code. Recently, an experiment reported the acceleration of gold ions up to 7 MeV/nucleon with a strong dependency of the charge-state distribution on target thickness and the detection of the highest charge states ${\text {Z}} \sim 72$. Our simulations using a developmental branch of EPOCH (4.18-Ionization) show that collisional ionization is the most important cause of charge states beyond Z = 51 up to He-like Au.

## Introduction

Following comprehensive studies of the laser-driven acceleration of light ions, mostly protons and carbon ions^1–4^, more attention is now being paid to the acceleration of heavy ions due to their potential applications in the studies of the astrophysical r-process nucleosynthesis of the heaviest elements in the Universe^5^.

Recently, an experiment^6^ performed at the PHELIX laser facility has reported the acceleration of gold (Au) ions up to 7 MeV/nucleon showing remarkable dependency of their charge-state distribution on target thickness and the observation of dominant charge states up to Z = 69 (Ne-like), well above the field-ionization (FI) prediction at such intensities. Previous simulations failed to account for these observations and predicted a sharp, dominant charge state of Z = 51 for 0.5 $\upmu$m Au foils (sub-ps laser pulse; ${\text {I}} \sim 2 \times 10^{20}$ W/cm^2^)^7^, which is not seen experimentally, along with negligible populations from other charge states (600 fs; ${\text {I}}= 5 \times 10^{20}$ W/cm^2^)^8,9^.

In the following, we present 2 dimensional (2D) simulations incorporating a new collisional ionization (CI) module and show for the first time that we are able to reproduce numerically high charge states up to Z = 77, in particular for thicker targets^6^. We believe that the CI process is the most important reason for increasing charge states well beyond Z = 51 up to Z = 77.

On the other hand, some discrepancies between the simulation model and experimental results remain, particularly for thicker targets: experiments show that the dominant charge state decreases with target thickness, whereas simulations imply that it should remain nearly constant. We address possible physical mechanisms which can cause such discrepancies.

## Results

To analyze gold ions, we selected those with E$\geqslant$ 1 MeV/nucleon within a forward 10$^{\circ }$ half-angle from the target normal since they are roughly collimated and are of interest for comparison to experimental results. The simulated Au macro-particles which satisfied these criteria were binned by their kinetic energy, using bins of size 1 MeV/nucleon. In EPOCH, the number of real particles represented by a macro-particle is given by the macro-particle weight, and in EPOCH2D, this weight is expressed as a number per unit length in the omitted dimension (here z dimension). Hence, to estimate the number of ions in 3D space, macro-particle weights have been multiplied by $\Delta$y$_{FWHM}$ = 4.3 $\upmu$m (our focal spot size used in the simulation; see “Methods” section.), which represents a typical transverse length-scale for this system. These modified macro-particle weights have been summed according to their charge states and affiliation to kinetic energy bins. This approximates the actual number of emitted ions with these energies.

**Figure 1:**
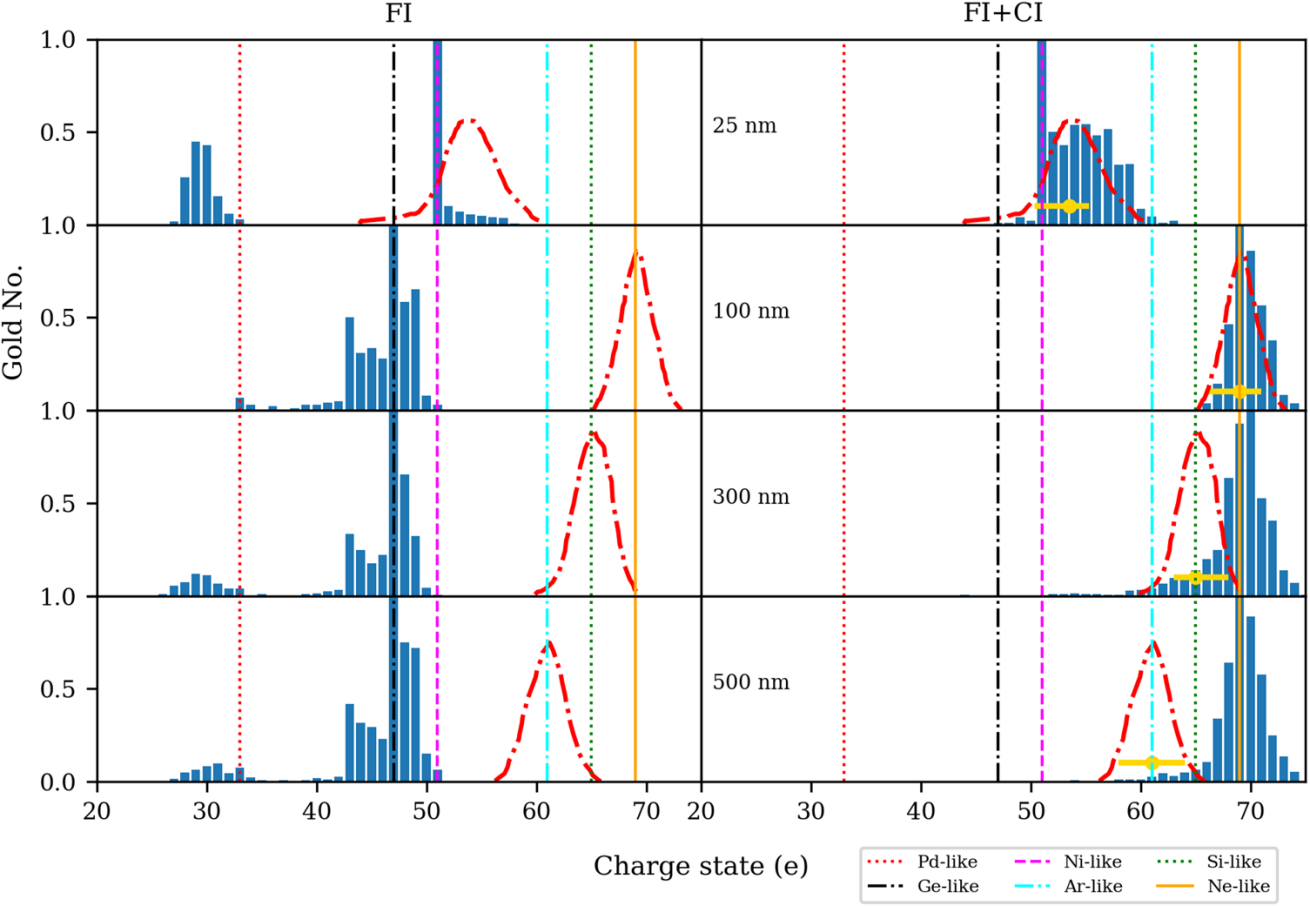
Simulation results of the charge state distributions of Au ions 2 ps after the interaction of a $4.1 \times 10^{20}$  W/cm^2^ laser pulse with 0.5 ps pulse duration with different gold foils with FI (left) and FI + CI (right). Only Au ions with E$\geqslant$ 1 MeV/nucleon within a forward 10$^{\circ }$ half-angle from the target normal are considered. Red dash-dotted curves and horizontal golden lines show experimental charge state distributions and error bars, respectively, as seen in the experiment (Fig. 9 of Ref.^6^). We can see a good agreement between simulations and experiments for thin targets, 25–100 nm. The only discrepancy is that for the 25 nm target, charge state 51 is much more populated in the simulation which is not seen in the experiment. For thick targets, charge state distributions statistically resemble ones that are seen in the experiment, but we see a down-shift of charge states in the experimental data toward lower ones, a trend that is not seen in the simulations.

Figure 1 shows the charge state distributions of Au ions 2 ps after the interaction of a $4.1 \times 10^{20}$ W/cm^2^ laser pulse with 0.5 ps pulse duration with different gold foil thicknesses with FI (left) and FI + CI (right).

In the case of FI, for ultra-thin targets, 10–25 nm, the dominant charge state is Z = 51 (Ni-like), while for thicker targets, 100–500 nm, it is Z = 47 (Ge-like).

In the case of FI + CI, for ultra-thin targets, 10–25 nm, the dominant charge state is still Z = 51 (Ni-like), while for thicker targets, 100–500 nm, it is now shifted to Z = 69 (Ne-like). Simulations vividly show that adding CI alters significantly the charge state distributions of Au ions. The most obvious effect is the population of charge states beyond Z = 51 up to Z = 77. In addition, all charge states below Z = 40 have been depopulated.

It is instructive to compare our simulation results with experimental data^6^ more quantitatively.

**Figure 2:**
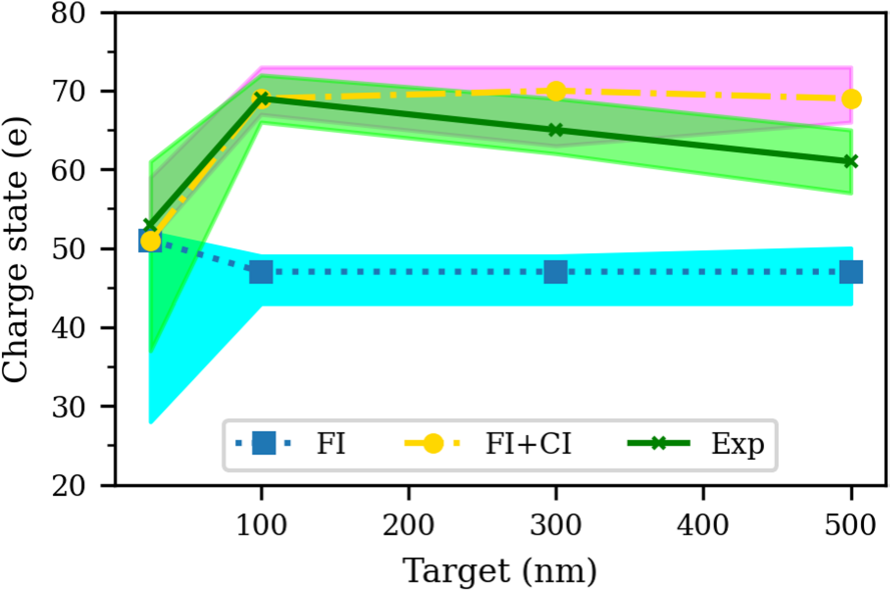
Simulation versus experimental results of the ranges (shaded areas), and dominant charge states of Au ions for different gold foil thicknesses. Shaded regions represent 10% of the bandwidth of the charge state distributions, i.e., all ionization levels that have number densities higher than 10% of that of the dominant charge state for each target thickness for FI simulation (cyan), FI + CI simulation (magenta), and experiment (green). Dominant charge states: FI simulation (blue square), FI + CI simulation (yellow circle), and experiment (green x). Only Au ions with E$\geqslant$ 1 MeV/nucleon within a forward 10$^{\circ }$ half-angle from the target normal are considered.

Figure 2 shows the minimum-to-maximum ranges, and the dominant charge states of Au ions for different gold foils thicknesses for the experiment and the simulations with FI and FI + CI.

Both for the simulations and the experiment, a clear maximum in the charge state distribution is observed. As can be seen for the ultra-thin target, 25 nm, both FI and FI + CI simulations show that the dominant charge state is Z = 51, which is very close to the experimental result, Z $\approx$ 53, considering the experimental uncertainties, Fig. 1. For thicker targets, 100–500 nm, the experimentally dominant charge states are close to the ones produced only with FI + CI simulations. For the 100 nm Au foil, simulation results match very well with the experimental data.

**Figure 3:**
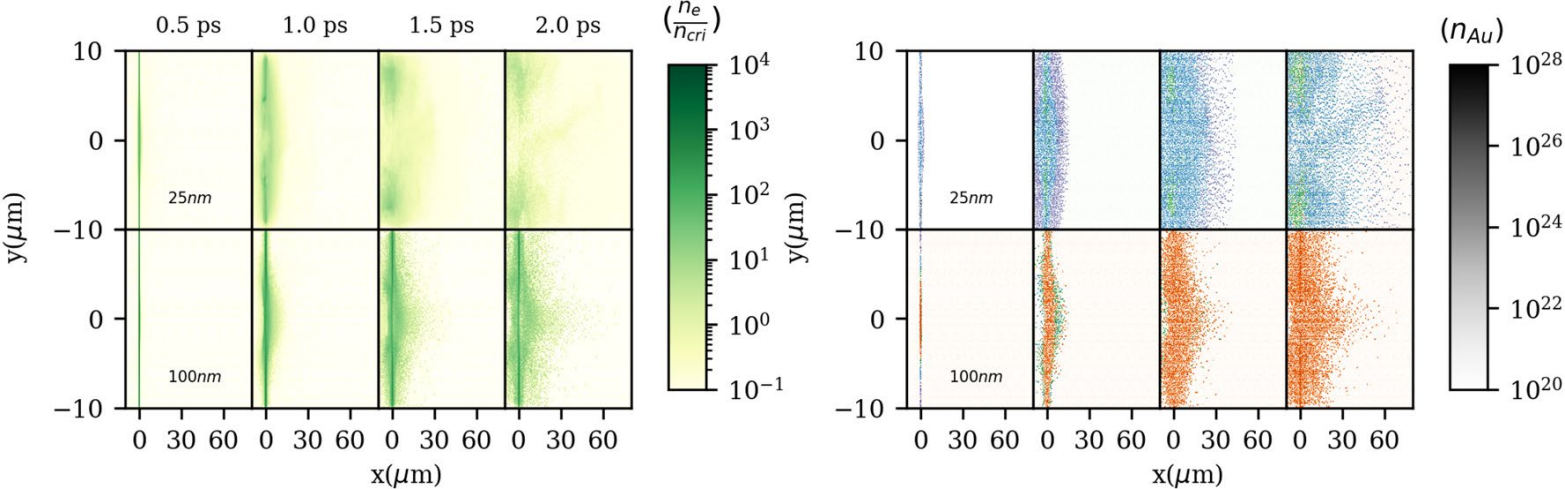
Left: electron densities normalized to n$_{cri}$. Right: Au ion densities for charge states of $\Delta$Z = 0–51 (purple), $\Delta$Z = 51–61 (blue), $\Delta$Z = 61–65 (green), and $\Delta$Z = 65–77 (orange). The colorbar indicates gold particle densities in m$^{-3}$ and is the same for all ionization levels. Results belong to the FI + CI simulations.

To address the question why for thick targets only FI + CI simulations give more accurate results, we plotted in Fig. 3 electron densities (normalized to the critical density of the laser, n$_{cri}$) and gold ion densities for 25, and 100 nm gold foils for FI + CI simulations.

For 25 nm gold foils, charge states of $\Delta$Z = 51–65 are observed, while for 100 nm foils a charge-state distribution of $\Delta$Z = 65–77 is found. Further analysis shows that 10–25 nm foils become transparent at t = 1.5–2 ps, while 100–500 nm foils remain overdense even after the interaction with the pulse. As thinner targets expand early and become transparent, the pulse easily penetrates the underdense plasma and FI can cause further ionization of gold ions. Moreover, as Au ions can accelerate early, the Au particle density decreases, and CI plays a minor role. For the 25 nm gold foil at t = 2 ps in Fig. 3, we can see the drop-off of the gold ion as well as electron particle densities in the laser propagation direction. For thicker targets, in contrast, as targets remain opaque, the pulse cannot penetrate the target and further ionization is provided mainly via collisions. That means Au ions are subject to more collisions with electrons inside the expanded target and CI plays a prevailing role. This can clarify why in Fig. 2 the experimentally dominant charge states for thinner targets are close to the FI simulations, while for thicker targets, 100–500 nm, they are better reproduced by FI + CI simulations. Also, for thick targets, ion-ion collisions can become more important and we will address this issue later.

## Discussion

The experiment^6^ shows that the 100 nm gold foil produces the highest dominant charge state, Z = 69. Seemingly, it is on the border between the FI-dominant and CI-dominant regimes : first, the target becomes semi-transparent and neutral atoms are ionized fast; then, CI increases the ion charge states: at t = 1 ps the minimum-to-maximum range of Au charge states is $\Delta$Z = 57–69, while at t = 2 ps it becomes $\Delta$Z = 66–77. Such a drastic and dynamic shift was only observed in the 100 nm case.

Previous numerical efforts^7–9^, even including both FI and CI in the simulations, contradict this observation and predicted that either the dominant charge state is Z = 51 or the number density of other charge states is negligible. One possible reason could be that for Refs.^8,9^ the Lotz formula was applied for estimating the CI cross section^10^, while in EPOCH the RBEB model^11^ is implemented. As the binding energies for highly charged Au ions were not available, ionization potentials were used in the Lotz formula which can be a source of significant discrepancies between simulations.

One remaining discrepancy between our simulation and experimental results is related to thick targets; experimentally dominant charge states are decreasing with increasing target thicknesses, while simulations show a semi-constant trend and that the minimum-to-maximum ranges of the charge states exceed the experimental ones. Several reasons can cause such discrepancies; firstly, EPOCH uses the RBEB model for CI cross section estimations for elements with Z $\ge$ 19, as discussed in the “Methods” section. However, this model relies on the *average kinetic energy of bound electrons* on each sub-shell, *U*, which must be evaluated using Dirac-Fock calculations^11^. Due to the computational expense of such a treatment, many PIC codes^12^ assume $U=B$, where *B* is the binding energy of electrons in the sub-shell. In Table VI of Kim et al.^11^, values of *U* and *B* are compared for xenon, which shows that $U=B$ is accurate in some sub-shells, but provides only an order of magnitude estimate in others. Typically U and B are in the same order of magnitude. As CI is the main process for thicker targets and depends on the cross section parameter, using less accurate cross section values might lead to unrealistically high ionization charge states. Following Fig. 5, cross sections do not seem very sensitive, even to massive changes in U. Hence, seemingly this parameter should not alter the results significantly.

We also note that ionizing Coulomb collisions will occur not only between electrons and atoms/ions, which we refer to as CI, but also between any two charged particles and in our case in particular between gold ions. Such inelastic collisions result in additional changes in the charge distribution and contribute additional free electrons to the plasma. For instance, the CI cross section of carbon ions for ionizing Au atoms is an order of magnitude higher than that of protons (Figs. 14-15 of Ref.^13^; Ref.^14^) and both are higher than that of electrons, which might mean contaminant ions can initiate the ionization of Au atoms in the same way as electrons. Including these *ion-ion collisions* in a full PIC simulation is currently not feasible, but we are working on implementing this feature.

**Figure 4:**
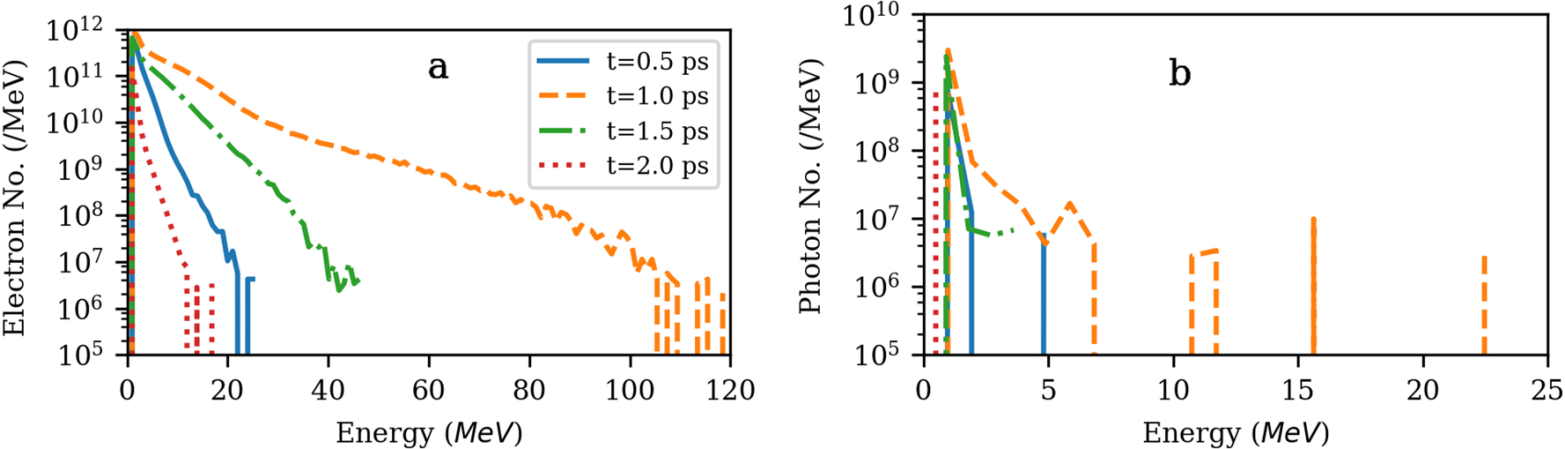
Temporal evolution of electron energy (**a**) and bremsstrahlung emission (**b**) due to the interaction of a $4.1 \times 10^{20}$ W/cm^2^ laser pulse with a 25 nm gold foil for the FI simulation.

Morover, energetic electrons with energies up to 120 MeV (according to our simulation), Fig. 4a, which can interact with the gold foil and produce X-rays via *bremsstrahlung* mechanism. We activated that routine for 25 nm gold target and observed X-rays up to 22.5 MeV, Fig. 4b. So if photo-ionization was sizable, one would have expected even higher charge states. But, bremsstrahlung energy loss scales with the background atom/ion number density while the cooling time is inversely proportional to it. Hence, bremsstrahlung radiation can be a major cooling mechanism for a high density plasma^15^; as thicker targets expand less, they have higher electron number densities compared to thinner ones. Therefore, stronger bremsstrahlung emissions are produced which radiate more efficiently and cool the plasma faster, and this could in turn influence the CI yield. Moreover, the effect of electron recirculation on bremsstrahlung radiations and emission source sizes is important and can be considered^16,17^

.

Following Fig. 4, the energies of the main electron population appear in the range of 1-5 MeV, but can reach up to 120 MeV, and bremsstrahlung emissions have energies mostly in the range of 1-2 MeV, but can acquire up to 22.5 MeV. It is instructive to compare the effectiveness of bremsstrahlung and CI ionization for that electron energy range and further ionizations via bremsstrahlung emissions with energies up to 22.5 MeV: Low energy electrons $\le$ 300 keV either ionize the target preferably from the M shell with cross sections of $\sigma \approx$ 10$^{-20}$ cm^2^ (Fig. 9 of Ref.^18^) or emit * forward low energy keV* bremsstrahlung radiation, $\sigma \approx$ 10$^{-21}$ cm^2^ , with energies $\le$ 200 keV (Figs. 4 and 9 of Ref.^19^). Such keV bremsstrahlung emissions produce low energy electrons via the *photoelectric* effect (Figs. 1-2 of Ref.^20^) which are then converted to *fluorescence emissions or Auger electrons* (Fig. 3 of Ref.^20^). Electrons with intermediate energies of 2–10 MeV still can strongly ionize the target from the M shell (Fig. 9 of Ref.^18^), however, bremsstrahlung cross sections are 5-6 orders of magnitude lower than the CI ones (Figs. 2-3 of Ref.^21^) which generate photons with energies of 0.1–10 MeV that preferably participate in *forward Compton scattering* (Fig. 5 of Ref.^20^). High energetic electrons with the energies of $\geqslant$10 MeV significantly ionize the target in the K-M shells, while bremsstrahlung emissions are strongly suppressed and if any photons are generated they have considerable energy, $\geqslant$10 MeV and participate mostly in *pair production*. Importantly, from the temporal evolution point of view, bremsstrahlung emission is very strong mainly near the peak of the laser pulse, while CI is significant considering its cross section even up to the end of the simulation, here 2ps.

Last but not least, the process that counteracts ionization is *recombination*, which is not accounted for in EPOCH and might be important. Recombination is mostly believed not to be important in a few ps after laser-target interaction^22^, but some studies have shown that the absence of that makes a clear difference to the final charge states of ions and to the temporal evolution of ionization^23,24^. Recombination of ions can occur via free or bound electrons. Ion recombination due to collisions with *bound* electrons proceeds via *charge exchange* with neutral or partially ionized atoms of the target^25,26^. *Free* electron-ion recombination becomes important when bound electron-ion recombination drops sharply at high plasma ionization degrees^27^ and consists of three processes: radiative recombination (RR), dielectronic recombination (DR), and three-body recombination (TBR)^14^ (inverse process to electron CI).

Laser plasma studies have shown that for the ionization process the *density effect* is much more important than the temperature dependence or screening effects of the target nucleus potential^27^. So, initially collisions are frequent due to a higher ionization rate in the dense regions^28^. The recombination rate drops in such dense regions and is a dominant process in a rarefied plasma in low temperature/density regions^27,29^. As time evolves the degree of ionization is determined via the mutual compensation of ionization and recombination processes which determine the final charge state distribution.

It is also important to consider the *temporal evolution* of the recombination of laser-generated plasma. Analysis shows that TBR occurs effectively in the dense plasma regions due to the high charge of ions. So, it occurs during the laser pulse, but, all auto-ionization states of the charged ions in a plasma are destroyed by the electric field, therefore, DR occurs in times considerably longer than the laser pulse duration in a rarefied plasma after pulse termination^25^. Moreover, the characteristic time of RR is in the order of a few hundred ps, which is very long compared to the fs laser pulse durations, hence it is less prominent than the DR and TBR mechanisms^25^.

From the *cross section point of view*, recombination rates of ions with bound electrons via charge exchange are typically 1-2 orders of magnitude higher than those of free electrons (Fig. 6 in Ref.^27^; Fig. 21 of Ref.^13^). Moreover, the recombination of high charge states of gold ions in heavy ion storage rings^30,31^ has much higher cross sections in the order of $\approx$ 10$^{-18}$ cm^2^ (Fig. 12 of Ref.^32^)–10$^{-20}$ cm^2^ (Fig. 1 of Ref.^33^), 1–100 times of the M shell CI cross sections ($\approx$ 10$^{-20}$ cm^2^; Fig. 9 of Ref.^18^). It means charge exchange and DR can be important recombination processes and compete strongly with CI in the dense and underdense regions of plasma, respectively. Importantly, the spectrum from an Au foil heated by a laser is similar to the one seen in a storage ring (Fig. 2 of Ref.^34^). A study about the recombination of tin ions has shown that RD, TBR, and finally RR are important (Fig. 5 of Ref.^35^).

Considering density effects, cross section values, and the temporal evolution of recombination of a laser-generated plasma, we first expect recombination of ions with bound electrons to occur via charge exchange in the cold, very dense regions of plasma due to their strong cross sections and also high numbers of bound electrons^27^. Then, free electron recombination is expected first via TBR, which is important at high densities and low temperatures below 1 keV^13,22,28^, relevant to laser-generated plasma, as mentioned in other studies^22^. Then, DR in the underdense region, which is specifically a dominant capture mechanism for very heavy highly charged ions^13,29^. Following Fig. 3 for the 100 nm target, we can see that the highest charge states of Au ions are generated in the underdense region, while lower ones are found within the high-density region, closer to the target. Finally, RR may lower Au charge states, though, is not very important^22^.

Accordingly, we believe the lack of recombination may explain the presence of very high charge states observed in our simulation in the charge-state range of Z = 65–77 for thick targets (Fig. 1), which is not seen experimentally ( Fig. 9 of Ref.^6^). Additionally, in the experiment (Fig. 4 of Ref.^6^), carbon and oxygen ions were detected as contaminants, whose recombination can reduce their charge especially that it is important also for light ions^36^ and for this reason, lower charge states are seen for these species. For protons, charge transfer with other atoms and electron-ion recombination in the high-density plasma near the target was unable to explain the neutralization, and instead, the recombination of copropagating electrons and ions far away from the target accounted for neutralization observed in the experiment^37^.

Importantly, if we consider the transition from charge state 50-> 51 via CI, which is a disputed issue between the simulation and experiment, and its opposite transition 51-> 50 via recombination, we might estimate that CI is more important. The reason is that the number and the cross sections of Au ions via CI is very high for this transition, while recombination of Au$^{51+}$->Au$^{50+}$ is an order of magnitude lower than that of Au$^{50+}$->Au$^{49+}$ ions (Figs. 1-2 of Ref.^38^; Fig. 4 of Ref.^39^). Hence, a single estimation of the efficiency of a recombination process for a specific ionization level may not only be inadequate, but can be misleading. Moreover, ions are not always directly ionized but also can be excited to a higher state which can then collide again and become further ionized, that strongly increases with the electron density, or decay back to a lower-bound state^27^. Accordingly, we need a PIC code that includes all relevant ionization, excitation, deexcitation, and recombination processes to cross-check the relative importance of different CI and recombination processes and to determine how, when, and where they become more important.

In conclusion, we observe in 2D PIC simulations that CI by free electrons can explain experimentally observed charge state distributions, including the high charge states well beyond what is expected from FI. But this requires a target that is thick enough to prevent substantial laser transmission and associated electron heating. For thinner targets that become transparent during the interaction, even for long pulses as considered here, FI can mostly explain the observed charge states where CI is insignificant, a conclusion which was drawn from experiments with much shorter laser pulses^40^. We, therefore, consider our study important, as it provides the first step towards a unified description of ionization in relativistically driven thin foil plasmas.

## Methods

For our simulations, we used the Particle-In-Cell (PIC) code EPOCH^41^. Targets were initially neutral solid gold foils with a density of 19.32 g/cm$^{3}$. Target thicknesses of 10, 25, 100, 300, and 500 nm were used with no contamination and can be considered as heated gold foils as used in the experiment^6^. The initial temperature of Au ions is zero.

Gaussian pulses (both temporally and spatially) with a wavelength of 1.053 $\upmu$m, duration of $\tau _{FWHM}$= 0.5 ps, and a waist of $\Delta$y$_{FWHM}$ = 4.3 $\upmu$m propagate normal to the target, in the x direction. Laser pulses enter the simulation box from the left side at x = -10 $\upmu$m and the polarization is oriented in the y direction. A solid gold foil is placed at x = 0. The phase of the pulses is zero at the moment of laser-target interaction. In the simulations, we used a Gaussian temporal shape function to have a smooth rising and falling of the laser pulse to avoid unrealistic effects due to the sharp cutoff of the laser pulse. Laser pulses are initiated with an intensity of $1 \times 10^{18}$ W/cm$^{2}$, and after 880 fs reach their peak intensity of $4.1 \times 10^{20}$ W/cm^2^. In all simulations, the left boundary radiates the laser pulse, while the right, upper and lower boundaries allow particles and radiation to leave the simulation box when they reach the boundaries.

Neither ion-ion nor atom-atom collisions were included in the simulations. We ran simulations with/without CI, but in all cases, field ionization (FI) was activated. FI is based on barrier-suppression ionization, tunneling, and multi-photon absorption^40^. A modified version of EPOCH was used to model CI, which is currently available on the 4.18-Ionisation branch^42^. The algorithm considered each cell individually, and iterated the charge states of macro-ions based on the estimated number of ionization events within the cell, over a simulation time-step, $\Delta t$. For ions of charge-state *Q*, the expected number of $Q+1$ ions, $N^{Q+1}$, created by an incident macro-electron of weight $W_e$ and speed $v_e$, over a time of $\Delta t$ is

1 $$\begin{aligned} N^{Q+1} = W_e (1 - \exp (n_i^Q \sigma ^Q v_e \Delta t)) \end{aligned}$$

where $n_i^Q$ is the number density of ions with charge-state *Q* within the cell^12^. The Relativistic Binary Encounter Bethe (RBEB) model was used for the electron impact ionization cross section $\sigma ^Q$^11^. Macro-ions of weight $W_i$ were chosen randomly in the cell, and those with $W_i < N^{Q+1}$ were ionised until the correct number of $Q+1$ ions were created. If $W_i > N^{Q+1}$, the ionisation was only performed with probability $N^{Q+1}/W_i$. Previous EPOCH versions used a modified RBEB (MRBEB) model^43^, although benchmarking has shown that MRBEB offered no significant improvement over RBEB, so RBEB was chosen here for consistency with other PIC codes^12^. Further corrections to the existing EPOCH models were implemented, including the sampling of ejected electron kinetic energies, and the inclusion of all bound electron contributions to $\sigma ^Q$.

**Figure 5:**
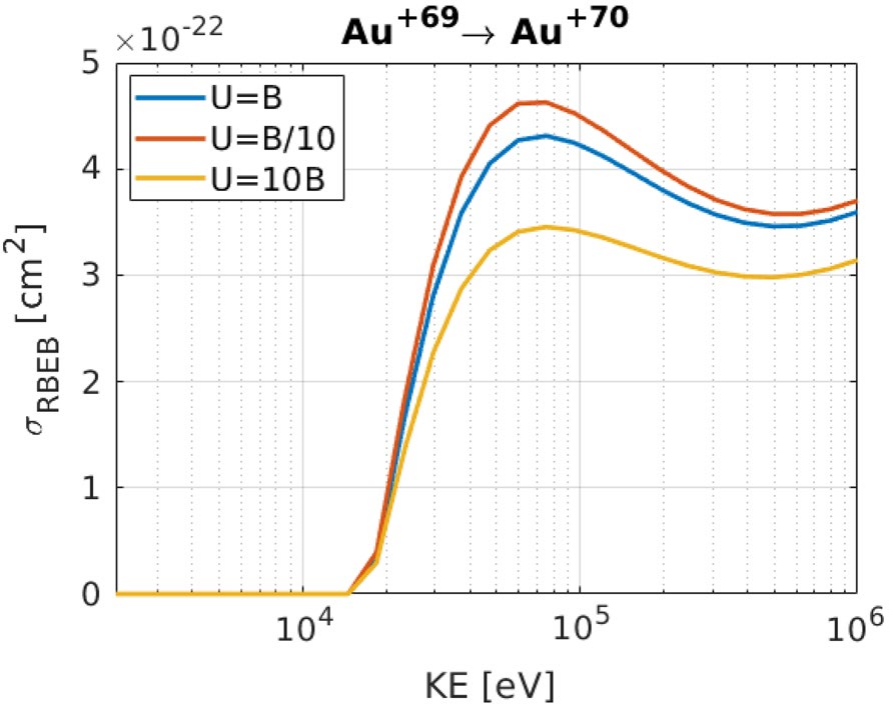
left: Cross sections of Au 69 $\rightarrow$ 70 transition for different U and B approximations.

Figure 5 shows the cross sections of Au 69 $\rightarrow$ 70 transitions for different U and B approximations.

For cases without CI, the simulation box has the dimensions of (-10, 90) $\upmu$m in x and $\pm 60$ $\upmu$m in the y direction. As the collision routine is very expensive computationally, in cases with CI, the y direction is reduced to $\pm 10$ $\upmu$m to be able to handle the simulations. For instance, the run times of CI simulations with reduced y dimension were six times longer than those of the FI simulations with larger y range with the same number of cores for all simulations. To check whether this affected the results, we performed a FI simulation with reduced y-dimension for a 100 nm foil and observed no difference in the results with those achieved with long y-dimension simulations. The total number of macro particles with and without CI is $3 \times 10^{4}$ and $18 \times 10^{4}$, respectively. The resolutions in the x-y directions are 6.67 nm and 10 nm, respectively. The numerical heating of 2D simulations until t = 2 ps is 10 eV/nucleon.

## Data Availability

The datasets used and/or analysed during the current study available from the corresponding author on reasonable request.
